# Quantitative Determination of a Series of Oxysterols by an Optimized LC-MS/MS Analysis in Different Tissue Types

**DOI:** 10.3390/ijms26010077

**Published:** 2024-12-25

**Authors:** Zhiting Guo, Huiyan Yu, Kexin Yang, Wenjing Feng, Miao Liu, Tao Wang, Rong Xiao

**Affiliations:** School of Public Health, Capital Medical University, Beijing 100069, China; guozhiting51@163.com (Z.G.); bjyuhuiyan@126.com (H.Y.); yangkx5989@163.com (K.Y.); 15810888862@163.com (W.F.); liumiao0330@163.com (M.L.); wangtao_930106@163.com (T.W.)

**Keywords:** oxysterol metabolism, liquid chromatography-tandem mass spectrometry, methyl tert-butyl ether, non-derivation, plasma, cerebral cortex, liver, mouse

## Abstract

Oxysterols, as metabolites of cholesterol, play a key role in cholesterol homeostasis, autophagosome formation, and regulation of immune responses. Disorders in oxysterol metabolism are closely related to the pathogenesis of neurodegenerative diseases. To systematically investigate the profound molecular regulatory mechanisms of neurodegenerative diseases, it is necessary to quantify oxysterols and their metabolites in central and peripheral biospecimens simultaneously and accurately. However, there are a lot of unsolved problems with the existing methods, such as the hindrance of applying a single method to different biological specimens or the challenge of simultaneous quantification due to differential groups on the ends of the oxysterol side chains. Herein, according to the physicochemical properties and structure of oxysterols, an optimized liquid chromatography-tandem mass spectrometry method for the quantification of oxysterols was established by optimizing the sample preparation process, chromatographic conditions, mobile phase pH, and solvent selection. Seven oxysterols were detected by this method, including 27-hydroxycholesterol, 7α-hydroxycholesterol, 7α,27-dihydroxycholesterol, 7-dehydrocholesterol, 7α-hydroxy-3-oxo-4-cholestenoic acid, 3-hydroxy-5-cholestenoic acid, and 24(S)-hydroxycholesterol. Non-derivatization extraction with methyl tert-butyl ether was used for different biospecimens, followed by simultaneous chromatographic separation of oxysterols on a phenyl hexyl column. By repeated validation, this method exhibited satisfactory linearity, precision, recovery, sensitivity, repeatability, and stability, and it was successfully applied to the detection of oxysterols in the plasma, cerebral cortex, and liver of mouse. In summary, our optimized method enables concurrent analysis and quantification of oxysterols and their metabolites in various biospecimens, presenting a broad range of applicability.

## 1. Introduction

**Figure 1:**
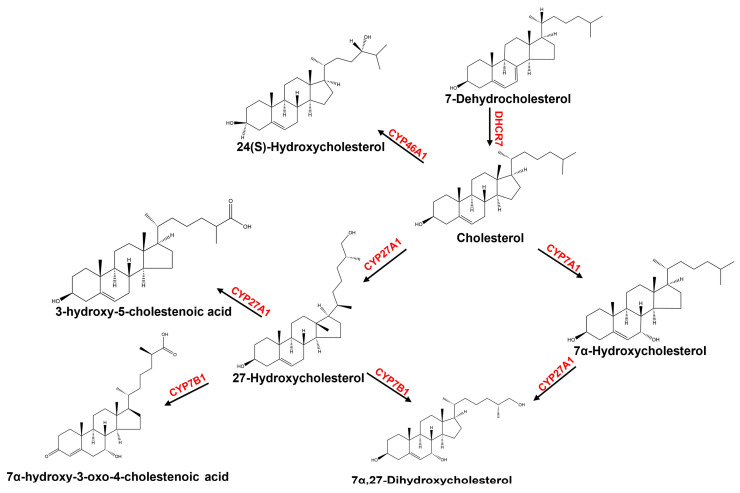
Structures of cholesterol, precursor, and metabolites. 7-DHC serves as a precursor to cholesterol. Once 27-hydroxylated, oxysterols become substrates for the enzymes CYP27A1 and CYP7B1 and continue with the process of oxidative metabolism. In addition, cholesterol is metabolized to 24(S)-OHC by CYP46A1.

Oxysterols, a family of structurally diversified bioactive lipid mediators, have been implicated in the pathophysiological processes of neurodegenerative diseases, such as Alzheimer’s disease (AD) and Parkinson’s disease (PD), significantly influencing the central nervous system function, immune cell response, and cell death or migration [1,2,3]. Oxysterols are formed via oxidation and hydroxylation of cholesterol hydrocarbon rings and side chains through cytochrome catalysis or ROS-mediated non-enzymatic reactions [4]. As a risk factor for neurodegenerative disease pathology [5,6], 27-hydroxycholesterol (27-OHC) is the predominant oxysterol in the periphery [7] and increases the level of amyloid-β 42 [8]. In contrast, 3-hydroxy-5-cholestenoic acid (27-CA), acting as a γ-secretase modulator, selectively reduces the level of amyloid-β 42 [9]. The contrasting effects of 27-OHC and 27-CA have attracted significant scientific interest due to their implications in neurodegenerative disease mechanisms. Therefore, we investigated the metabolic network of 27-OHC to gain insights into its role in maintaining metabolic homeostasis. 27-OHC in the brain can be converted into 7α-hydroxy-3-oxo-4-cholestenoic acid (7-HOCA) (Figure 1) [10,11,12]. In addition, 7α-hydroxycholesterol (7α-OHC) and 7α,27-dihydroxycholesterol (7α,27-diOHC), both of which are associated with 27-OHC, significantly influence the progression of neurodegenerative diseases [12,13,14,15,16,17] and are also involved in the regulation of neuroinflammatory responses [13]. Given the inextricable and subtle association of the metabolic network of 27-OHC with neurodegenerative diseases [10], targeting metabolic network homeostasis disruptions may provide new medical strategies for disease prevention, biomarker threshold establishment, and the development of new therapies. In contrast to 27-OHC, 24(S)-hydroxycholesterol (24(S)-OHC) acts as a protective agent in the brain [8]. It is also a potential biomarker in plasma analysis for neurodegenerative diseases [18]. Furthermore, the level of plasma 24(S)-OHC is a key indicator of disease burden and the number of metabolically active neurons in neurodegenerative diseases [19]. Therefore, it is necessary to establish a stable, simplistic, and rapid quantitative method to quantify the metabolic network of 27-OHC and 24(S)-OHC simultaneously [20,21,22,23], so as to pave the way for the quantitative study of metabolic homeostasis.

Recent advancements in chromatography technology have driven lipidomics, enabling detailed characterization of lipids [24,25,26]. Notably, high-performance liquid chromatography (HPLC) has become the dominant technique among different chromatographic techniques. Tandem mass spectrometry provides structural information on lipid molecules, enabling accurate qualification and quantification [27]. Due to the complexity of the intrinsic chemical structure of the human lipid molecule, electrospray ionization mass spectrometry is commonly used, including shotgun lipidomics, liquid chromatography–mass spectrometry, thin-layer chromatography, gas chromatography (GC), matrix-assisted laser desorption ionization-time-of-flight and imaging lipidomics, among others [28]. 

There is extensive research on the quantification of oxysterols at present [4,29,30,31,32,33,34,35]. This includes derivatization and non-derivatization methods, with a primary focus on LC-MS/MS and GC techniques. The present research landscape indicates that existing methodologies face challenges in simultaneously quantifying oxysterols and their metabolites, primarily due to their structural variations. Due to the naturally low abundance and matrix differences, these factors pose significant barriers to their detection and quantification using analytical techniques. At present, derivatization methods are the mainstream option, but their complexity and time-consuming nature hinder their application in large-scale sample detection. GC is known for its excellent chromatographic separation. However, GC-based methods are hampered by long run times and large sample volumes [19,31], which prevent them from becoming a routine method for oxysterol quantification. On the other hand, LC-MS/MS was eventually applied to our research based on its reliability in the development of mass spectrometry and superior flexibility compared to immunoassays [36].

In this study, we aim to develop an optimized and validated LC-MS/MS method for the simultaneous and rapid quantification of oxysterols and their metabolites in the plasma, cerebral cortices, and livers of mice without derivatization. The substances detected in this experiment include seven oxysterols, 27-OHC, 7α-OHC, 7α,27-diOHC, 7-dehydrocholesterol (7-DHC), 7-HOCA, 27-CA, 24(S)-OHC, and two internal standards (ISs), 27-hydroxycholesterol-26,26,26,27,27-d5(27-OHC-D5) and 24-hydroxycholesterol-25,26,26,26,27,27,27-d7 (24-OHC-D7). The advantages of the present method are that the same extraction system can be used in different biospecimens, and multiple alcohols and cholestenoic acids in oxysterols can be detected simultaneously. By studying the metabolic network of 27-OHC and 24(S)-OHC, the localization of oxysterols from both central and peripheral sources can be further explored, and the pathological and molecular mechanisms of neurodegenerative diseases can be investigated in depth and systematically, so as to provide new medical strategies for the prevention of neurodegenerative diseases.

## 2. Results

### 2.1. Parameter Optimization

We optimized the LC-MS/MS conditions to improve chromatographic separation and increase the stability of the analysis. To improve the separation and detection of nonpolar compounds, we optimized the source temperature, nebulizer current, flow rate, Gas 1, left temperature / right temperature, retention time, and curtain gas. By adding or subtracting the parameter values for various conditions within a reasonable range, the peak area of the oxysterols was changed. We tried to adjust the value of one parameter while keeping the others constant. Considering stability and sensitivity, we identified the optimal LC-MS/MS parameters based on the peak heights of each oxysterol, as listed in Table 1. The optimized method can be used to measure oxysterols we have tested effectively.

### 2.2. Mobile Phase

**Figure 2:**
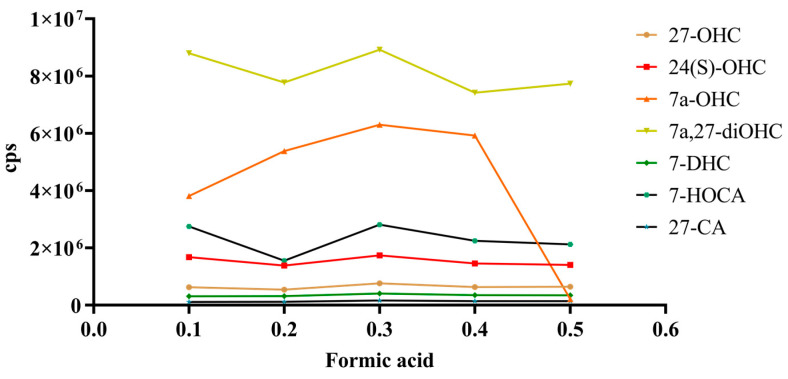
Chromatographic peaks of mobile phase A at varying formic acid concentrations. As the content of FA in mobile phase A rose from 0.1% to 0.5%, the line chart exhibited an approximate W-shaped distribution. It reached the highest at water with 0.3% (*v*/*v*) FA.

The effects of different doses of formic acid (FA) added to mobile phase A on the peak area of oxysterols were investigated (Figure 2). By comparing the peak area and peak height of each oxysterol, we found that water with 0.3% (*v*/*v*) FA produced optimal peak shapes and sensitivity. As the proportion of FA increased, we found that baseline noise was also increasing. Therefore, 0.3% FA aqueous solution was ultimately selected as mobile phase A.

### 2.3. Linearity and Sensitivity

**Figure 3:**
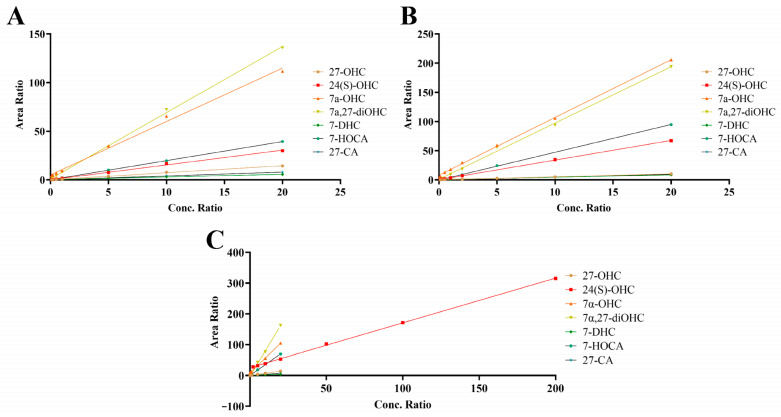
The regression curves of oxysterols. 27-OHC, 27-hydroxycholesterol; 24(S)-OHC, 24(S)-hydroxycholesterol; 7α-OHC, 7α-hydroxycholesterol; 7α,27-diOHC, 7α,27-dihydroxycholesterol; 7-DHC, 7-dehydrocholesterol; 27-CA, 3-hydroxy-5-cholestenoic acid; 7-HOCA, 7α-hydroxy-3-oxo-4-cholestenoic acid. (**A**) The figure showed the regression curves of seven oxysterols in BSA. (**B**) The figure showed the regression curves of seven oxysterols in the liver. (**C**) The figure showed the regression curves of seven oxysterols in the cortex homogenate. Due to the high concentration of 24(S)-OHC in the cortex, the regression curve of 24(S)-OHC was particularly prominent.

The detection range was determined according to the clinical detection requirements of seven oxysterols, and the concentration gradient was established. The standard method for LC-MS/MS quantitation involves using the peak area ratio, which is the ratio of the analyte peak area to the IS peak area. The standard regression curve was obtained by fitting a regression between the peak area ratio and concentration. The regression curve and its equation are shown in Figure 3 and Table 2. The standard curve showed satisfactory linearity, and the coefficient of determination (R^2^) exceeded 0.995. Meanwhile, the lower limit of detection (LLOD) and lower limit of quantification (LLOQ) are listed in Table 2.

### 2.4. Precision

Based on three QC sample levels, the precisions of oxysterols are listed in Table 3. The intra-day imprecisions were all within the range of 0.47% and 14.05%. The inter-day imprecisions were all within the range of 1.54% and 9.96%. The results showed satisfactory precision of the present method.

### 2.5. Recovery

The recovery of the present method was confirmed through the analysis of low-, mid-, and high-QC samples. Table 4 provides a summary of the actual recoveries. In the plasmas, cerebral cortices, and liver, the recoveries for low, medium, and high QC samples remained within the range of 88.47% to 112.32%.

### 2.6. Matrix Effect

Unbiased observation of metabolite levels is essential for targeted metabolite quantification and mass spectrometry [37]. Matrix effects are a well-known measure of bias observation. Table 5 shows that the ME% of oxysterols for QC samples ranges from 85% to 115%. The results showed that the plasmas, cerebral cortices, and livers of mice did not have a significant effect on the quantitative results of oxysterols in the non-derivatization extraction of MTBE, and there was no obvious observation bias.

### 2.7. Repeatability and Stability

The repeatability and stability of oxysterols are detailed in Table 6. Following the detection of six repeated extremely low concentration samples, the coefficients of variation (CVs) for plasmas, cerebral cortices, and liver were consistently below 15%. The results indicated that the present method possessed satisfactory repeatability. The CVs of the samples were below 15% after 0, 4, 12, and 24 h measurements at 4 °C and 25 °C. Stability results indicated that the needs of clinical and laboratory applications can be met.

### 2.8. Detection of Biological Samples

**Figure 4:**
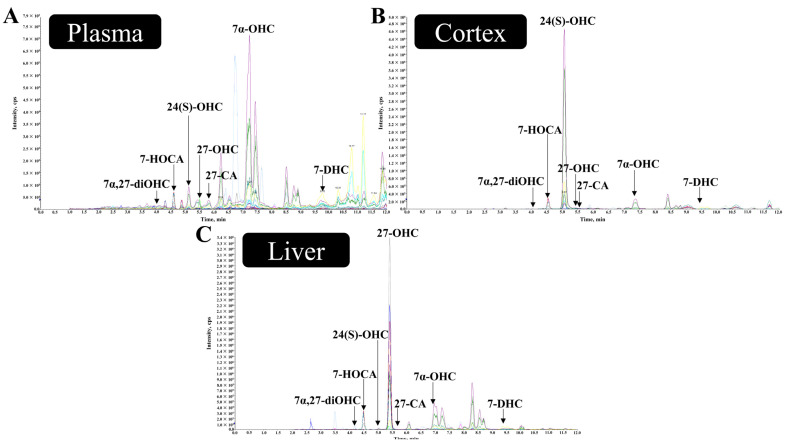
Chromatograms in mouse plasma, cerebral cortex, and liver. 27-OHC, 27-hydroxycholesterol; 24(S)-OHC, 24(S)-hydroxycholesterol; 7α-OHC, 7α-hydroxycholesterol; 7α,27-diOHC, 7α,27-dihydroxycholesterol; 7-DHC, 7-dehydrocholesterol; 27-CA, 3-hydroxy-5-cholestenoic acid; 7-HOCA, 7α-hydroxy-3-oxo-4-cholestenoic acid. (**A**) The figure shows the chromatogram of mouse plasma. (**B**) The figure shows the chromatogram of the mouse cerebral cortex. (**C**) The figure shows the chromatogram of the mouse liver.

To verify the feasibility of the established method, we randomly selected plasmas, cerebral cortices, and livers from twenty C57BL/6J wild mice. Using the extraction method mentioned above, the concentration of oxysterols in biological samples was measured. The results are shown in Table 7 and Table 8 and Figure 4. The results showed a low level of 7-HOCA in the cortices because of its functional positioning and metabolic route [38]. In addition, we found that the concentration of 7a,27-diOHC in the plasmas was extremely low. Since cholesterol cannot cross the BBB, 24(S)-OHC accumulates in large quantities in the brain.

## 3. Discussion

Given the importance of oxysterol homeostasis, it is imperative to develop LC-MS/MS analysis and sample preparation methods based on the physicochemical properties of these metabolites in order to quantify the metabolic network of 27-OHC and 24(S)-OHC in plasma, cerebral cortex, and liver simultaneously. However, there are few studies that have been able to quantify oxysterols and their metabolites in different types of biospecimens simultaneously using the same method at present. For the non-polar properties of oxysterols, the researchers’ main focus was drawn to derivatization, but it has inherent drawbacks, such as operational difficulties, poor reproducibility, and high cost. The presence of a large number of isomers and cholestenoic acids in oxysterol metabolites makes differentiation by derivatization more difficult to implement. Hence, we developed and validated a non-derivatized LC-MS/MS method capable of measuring seven oxysterols simultaneously from just 100 µL of plasma or 30 mg of cerebral cortex/liver. MRM mode using triple quadrupole mass spectrometry was used in the present method because of its sensitive and selective quantitative performance and reliable monitoring of low lipid concentrations [39]. By optimizing the collision energy and other parameters for each analyte, MRM settings were conducted for seven oxysterols. LC-MS/MS was also suitable for high-throughput analysis: a batch of 100 samples can be analyzed within 24 h, and a single LC-MS/MS detection of seven oxysterols took only about 12 min.

### 3.1. Optimization of Mass Spectrometry Conditions

In the process of optimizing the mass spectrometry conditions, we detected the adduct ions. Adduct ions are formed by the interaction of a precursor ion with one or more atoms or molecules, and the resulting ion contains all the atomic components of the precursor ion and the additional atoms of the associated atom or molecule [40]. We performed simultaneous mass spectrometry detection of the ion pairs of seven oxysterols to compare the precursor ions of the ammoniated adduct and sodium adduct (traces of sodium formate or ammonium acetate were present in the solvent system of both). Precursor ions involve [M+H]^+^, [M+2H]^2+^, [M+H-H_2_O]^+^, [M+Na]^+^, [M+2Na]^2+^, [M+NH_4_]^+^, and [M+N_2_H_8_]^2+^. After comparing the peak shape of the precursor ions, it was found that the detection rate and peak area of the sodium adduct and ammoniated adduct were lower. The peaks were hidden below abundant baseline noise. And there was ghost peak interference, which had an impact on the final result. This was consistent with previous research [41]. Accordingly, we compared the effects of ESI and atmospheric pressure chemical ionization (APCI) on the ionization efficiency of the analyte. ESI has higher sensitivity, accuracy, and ionization efficiency than APCI [42]. This conclusion was verified by our controlled experiments. Although the APCI exhibits reproducible MRM transitions, ESI is ultimately suitable for analysis due to the advantages of short analysis time, high efficiency, low sample consumption, and low solvent consumption [43]. In addition, we evaluated the efficiency of the ESI in positive/negative ionization. Due to the presence of at least one hydroxyl functional group in the oxysterols, satisfactory ionization was finally achieved in positive ionization mode. Although many researchers have described acetonitrile as having satisfactory solubility [37,38,44,45], our experiments showed that the solubility of oxysterols in acetonitrile was not improved, so it was not the optimal choice for redissolving in the present method. Methanol has shown good applicability in dissolving oxysterols [46,47,48]. Thus, we made additional attempts in this regard. We added the same volume of deionized water to methanol to increase solubility, but pure methanol still showed the most satisfactory results. Furthermore, we compared the extraction capacity of the MTBE system between single and double extractions. A single extraction was used in the present method, as double extraction increased the workload based on no further increase in sensitivity and peak height. It was found that the addition of FA to the mobile phase to adjust the mobile phase into an acidic environment could increase the sensitivity of the assay [49]. Therefore, we experimented with the optimal concentration of FA in the mobile phase, and the results revealed the best peak area and sensitivity of oxysterols when 0.3% formic acid was added to mobile phase A (Figure 2).

### 3.2. Optimization of Pretreatment and Extraction Method

The extraction solvent and extraction method were optimized to improve the ionization efficiency of the ESI. In order to purify or characterize oxysterols in vivo, it is important to choose an effective method to disrupt cells or tissues, and a variety of homogenization modalities have been reported [4,50,51]. Considering the water solubility of hydroxycholesterol and cholestenoic acid, we tested five homogenization techniques for cerebral cortex and liver tissues, namely MeOH: normal saline (1:1, *v*/*v*), MeOH: chloroform (1:1, *v*/*v*), and MeOH: MTBE: normal saline (3:10:2.5, *v*/*v*). The results of these five methods, despite slight differences, were not statistically different. It may be due to the fact that after homogenizing the tissue, we collect it all into a centrifuge tube for the next step, without generating additional consumption. On the downside, the disadvantage is that once the organic solvent is added during the homogenization process, it will cause the tissue morphology to change to a spherical shape with a smooth surface, and it is not easy to homogenize completely. Multiple LC-MS/MS methods for the extraction of oxysterols from biospecimens of mice have been previously described [4,16,31,52,53,54,55,56,57]. Oxysterols were extracted by liquid chromatography–mass spectrometry solid phase extraction (SPE) [4] to separate them from other components. Ideally, oxysterols can be completely separated from the sample by SPE, as this will simplify subsequent instrumental analysis. However, SPE does not have the intrinsic resolution or reproducibility to separate and quantify oxysterols from all other components [58]. In addition, some researchers used a solution containing hexane and isopropanol (99:1, *v*/*v*) for extraction, and added antioxidants (butylated hydroxytoluene and potassium hydroxide) and metal chelator ethylene diamine tetraacetic acid [11]. After repeated experiments, it was found that antioxidants react chemically with cholestenoic acids, resulting in lower detection values. Hexane–isopropanol extraction was also relatively inefficient, and the procedure was redundant. Excessive oxysterol was lost during the experiment. If an extraction system containing dichloromethane, methanol, and water (86:14:1, *v*/*v*/*v*) was used [58], the difference in density would lead to the formation of a two-phase partition system. When collecting the dichloromethane fraction, the needle of the glass pipette passed through a layer of non-extractable and insoluble matrix to reach the dichloromethane fraction, which was usually located at the interface between the water/methanol and dichloromethane phases. It was difficult to aspirate the lower organic phase, and it will aspirate part of the upper phase inevitably, resulting in more impurities adhering to the centrifuge tube wall after vacuum concentration, which may block the ion source or column. The use of a solution containing methanol and isopropanol (1:1, *v*/*v*) to extract oxysterols [52] had problems such as tailing peaks. In addition, the known carcinogenicity of chloroform poses considerable health risks to laboratory personnel [59]. Ultimately, MTBE is suitable for research due to its excellent extraction capability. Considering the ability of methanol to reduce the hydrophobic effect between sterol molecules, reduce the hydrogen bonding and electrostatic interaction between membrane lipids and membrane proteins, and precipitate proteins [60,61,62], we used an optimized mixture of MTBE, methanol, and water (10:3:2.5, *v*/*v*/*v*) as the extraction solvent system. The mixture in this ratio is miscible and forms a phase. Biological samples, such as blood, cerebral cortex, and liver, are homogenized in this mixture to extract oxysterols.

Although derivatization has been shown to offer more characteristic structural information, improve the analytical performance, and provide one-to-one chemical isotope labeling IS based on the isotope derivatization regent in quantitative analysis [63,64], we need to consider factors such as reaction conditions, derivatization efficiency, selectivity, and stability of derivatives. Among our analytes, alcohols and acids were present in the same system and were difficult to determine by the same derivatization method. Derivatization is time-consuming and its process is complex. A researcher had proposed picolinyl ester derivatization [54], but its separation time of nearly 40 min makes it difficult to detect large sample batches. In addition, another researcher proposed a two-step derivatization method for hydrazination by (2-hydrazinyl-2-oxoethyl) trimethylammonium chloride (Girard reagent), but they also pointed out that it was difficult to separate cholestenoic acids from oxysterols completely [65]. Furthermore, there are ghost peaks generated by keto groups in the background, interfering with the analysis of the target peak. During the process of derivatization, depletion of the substance to be measured can also easily occur. Therefore, we have discarded the derivatized sample preparation method and opted for a non-derivatization process with a lower loss rate.

### 3.3. Application

To test the feasibility of the method, we applied it to C57BL/6J mice. Based on the method, we tested twenty mouse plasmas, cerebral cortex homogenates, and liver homogenates (Table 7 and Table 8). The results showed excellent separation efficiency and reproducibility. The homeostasis of 27-OHC and 24(S)-OHC in production and secretion may reflect the dual role of oxysterols, which act as carriers of cholesterol transport and signal transmitters for nerve cells [66]. Considering the important role of 27-OHC and 24(S)-OHC in the progression of neurodegenerative diseases [67], we focused on them. Serum and cerebral cortex 27-OHC concentrations in 9-month-old mice were approximately 10.7 and 0.4 ng/mL by LC-MS/MS, respectively, which was consistent with what was described in previous studies [68,69]. Coincidentally, Hepatic 27-OHC is age-dependent, rising with age [4,70]. In a previous study, the researchers proposed that the level of 27-OHC in the liver of mice aged 2–5 months was about 0.2 ng/mg [71], our study found that the level of 27-OHC in the liver of 9-month-old mice was about 0.5 ng/mg, while the concentration of 24(S)-OHC was consistent with this work. The difference in the concentration of 27-OHC in the results may be due to using mice aged at different months. Unlike 27-OHC, plasma 24(S)-OHC peaks at about 75 ng/mL 12–15 days after birth, and then begins to decline [72]. Several studies have reported that the concentration of 24(S)-OHC in the mouse cerebral cortex is approximately 30–60 ng/mg [73,74], which is similar to the level detected in our work. In the liver, 24(S)-OHC was almost undetectable (<0.1 ng/mL) [71], possibly due to the fact that cholesterol 24-hydroxylase is expressed primarily in the brain rather than in the liver [75].

In particular, 7α,27-diOHC was not detected in plasmas. It is speculated that this may be due to the fact that the plasma of 7α,27-diOHC is below the lower limit of detection. This is consistent with a recent study [76]. The concentration of 7α-OHC was also similar to that found in other studies [76,77]. In addition, 7-HOCA and 27-CA are predominantly found in the circulation and are, therefore, almost undetected in the cerebral cortex [9,78,79]. Consistent with the work of Klementina [80], we detected that the level of 7-DHC in the mouse cerebral cortex and liver was approximately 7.4 ng/mg and 2.8 ng/mg tissue weight. Interestingly, we noted that the levels of 7-DHC varied widely between studies [71,81,82], suggesting that this may be related to the duration of light exposure to the mice’s growth activities, the amount of vitamin D in their diets, and the duration of their activities. Therefore, as described in the section “Sample Pretreatment and Oxysterols Extraction”, we performed our best to control as much as possible for differences in sample processing conditions and survival conditions between different mouse individuals to minimize the effect of confounding factors on 7-DHC levels.

In conclusion, a comprehensive LC-MS/MS-based profiling method has been developed to enable the stable, simplistic, and simultaneous quantification of oxysterols and their metabolites in a single analysis, and has been successfully applied to the plasma, cerebral cortex, and liver of mice, providing an opportunity for in-depth and systematic study of the pathological mechanism of neurodegenerative diseases and the metabolic homeostasis of oxysterols in the central and peripheral regions.

## 4. Materials and Methods

### 4.1. Sample Collection

The plasma, cerebral cortex, and liver of the mouse were derived from 9-month-old C57BL/6J wild mice (Beijing SPF Biotechnology Co., Ltd., Beijing, China). All steps and operations were conducted according to the recommendations in the Guide for the Care and Use of Laboratory Animals of the National Institutes of Health and were approved by the ethics committee of Capital Medical University (AEEI-2022-007). Mice were housed in a temperature- and light-controlled, specific pathogen-free environment with free access to water and feed. After blood collection, whole blood samples were centrifuged at 12,000× *g* rpm for 15 min. The plasma supernatant was collected, immediately placed on dry ice, and stored at −80 °C until analysis. The cerebral cortex and liver were stored at −80 °C immediately after removal until analysis.

### 4.2. Reagents and Equipment

7α-OHC and 7-HOCA were purchased from Avanti Polar Lipids Inc. (Alabaster, AL, USA). 27-CA was purchased from TRC Companies, Inc. (Houston, TX, USA). 7α,27-diOHC was purchased from Sigma-Aldrich (Diegem, Belgium). 27-OHC was purchased from Santa Cruz Biotechnology, Inc. (Santa Cruz, CA, USA). 27-OHC-D5 and 24-OHC-D7 were purchased from Medical Isotopes, Inc. (Pelham, NH, USA). 24(S)-OHC was purchased from Abcam Limited. (Cambridge, UK). 7-DHC was purchased from Shanghai Macklin Biochemical Co., Ltd. (Shanghai, China). Methanol was purchased from Thermo Fisher Scientific Inc. (Fair Lawn, NJ, USA). Ultrapure Water Purification System was purchased from SUEZ Group (Preston PR2 2NQ, UK). Formic acid was purchased from Aladdin Scientific Corp. (West Palm Beach, FL, USA). Equipment for LC-MS/MS, chromatographic column was purchased from Agilent Technologies, Inc. (Santa Clara, CA, USA), and mass spectrometer SCIEX Triple Quad™ 6500 was purchased from AB Sciex Pte. Ltd. (Framingham, MA, USA). Liquid chromatography system 1290 Infinity was purchased from Agilent Technologies, Inc. (Santa Clara, CA, USA). Methyl tert-butyl ether (MTBE) was purchased from J&K Scientific Ltd. (Chaoyang District, Beijing, China). β-Cyclodextrin was purchased from Cayman Chemical (Ann Arbor, MI, USA). Bovine Plasma Albumin (BSA) was purchased from Solarbio Life Science (Tongzhou District, Beijing, China). Table refrigerated centrifuge was purchased from Sigma-Aldrich (Diegem, Belgium). ThermoMixer^®^C, purchased from the Eppendorf Group (Hamburg, Germany), was utilized for vortex mixing. The multipurpose shaker was purchased from Kylin-Bell Lab Instruments Co., Ltd. (Haimen City, China). Vortex Shaker QL-901 was purchased from Kylin-Bell Lab Instruments Co., Ltd. (Haimen City, China). Concentrator Plus was purchased from Eppendorf Group (Hamburg, Germany) for vacuum concentration. All solvents were HPLC grade or higher and all chemicals were ACS grade or higher.

### 4.3. Working Solutions, Concentration Gradient, and Quality Control Samples

Working solutions were prepared through serial dilution of seven standard stock solutions (1 mg/mL) with methanol. IS working solutions for plasmas/liver homogenates or cortex homogenates (1 μg/mL of 27-OHC-D5 or 24-OHC-D7) were also prepared in methanol. IS was used to correct variances during the entire analytical process, including sample preparation, chromatographic separation, and mass spectrometry. By correcting variances, IS enables more accurate quantitation [83]. Given the endogenous oxysterols in plasma, cerebral cortex, and liver, the concentration gradients of oxysterols were prepared by using 5% BSA and cortex/liver homogenate (diluted tenfold) to simulate the substrate milieu [67,84,85,86]. We prepared several gradients of different concentrations (STD1–STD7) covering physiological ranges by adding working solutions to the alternative matrices, followed by stepwise dilution. The concentration gradients of oxysterols are listed in Table 9. 5% BSA and cortex/liver homogenate (diluted tenfold) were used as blank controls. Quality control samples were prepared by adding different concentrations of working solutions to the appropriate matrix. The QC samples for the metabolic network of 27-OHC and 24(S)-OHC were set to low-quality control (LQC, 10 ng/mL), mid-quality control (MQC, 100 ng/mL), and high-quality control (HQC, 800 ng/mL). Especially, given the high level of 24(S)-OHC in the cerebral cortex, the concentration of LQC was set to 250 ng/mL, the concentration of MQC was set to 1 μg/mL, and the concentration of HQC was set to 4 μg/mL.

### 4.4. Sample Pretreatment and Oxysterols Extraction

**Figure 5:**
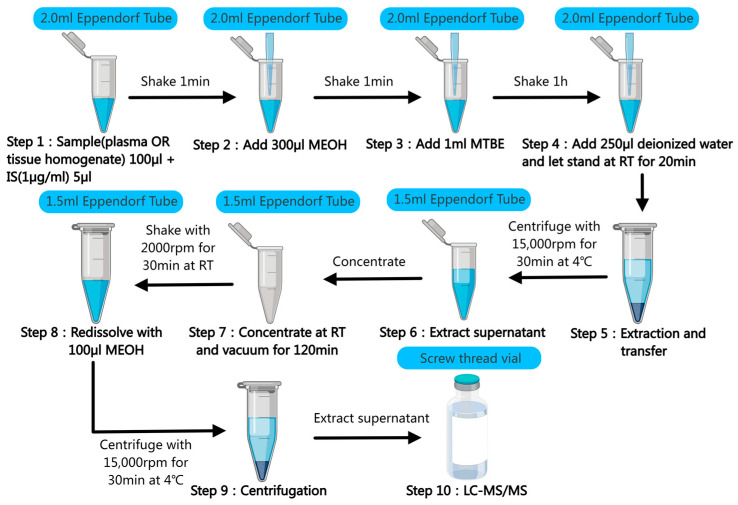
The procedure of the extraction for oxysterols in biological matrix. First, when adding the IS to the centrifuge tube, it is important to ensure that the pipette tips are fully submerged below the liquid level in order to avoid inadequate volume and inaccurate results. Second, mixing is required at each step of the working fluid addition to ensure uniformity. Third, centrifugation is kept for as long as possible for 30 min to ensure precipitation of impurities in the brain and blood. Finally, when aspirating the supernatant, it is necessary to avoid aspirating to the lower aqueous phase as much as possible.

For the cerebral cortex or liver, about 30 mg was taken and 240 μL of normal saline was added. The tissue homogenate was collected completely. For the plasma, the frozen sample (stored at −80 °C) was thawed on ice. To guarantee homogeneity, the sample was mixed with a vortex shaker for 1 min. A total of 100 μL of plasma or cortex/liver homogenate was added into a 2 mL centrifuge tube. As IS, 5 μL of 27-OHC-D5 (1 μg/mL) was added to the plasma or liver homogenate, and 5 μL of 24-OHC-D7 (1 μg/mL) was added to the cortex homogenate, respectively, and mixed thoroughly. Then, 300 μL MeOH was added and blended in a vortex for 1 min. Subsequently, 1 mL MTBE was added and shaken at room temperature for 1 h (42 rpm). Finally, 250 μL deionized water was added and mixed, followed by standing for 20 min at room temperature. Under the conditions of 15,000 rpm and 4 °C, the table refrigerated centrifuge was centrifuged for 30 min. The upper organic phase of 800 μL was placed in a 1.5 mL centrifuge tube in V-HV mode and concentrated by vacuum at normal temperature. Due to the addition of deionized water, it is necessary to pay attention to the bottom of the centrifuge tube for the residue of the aqueous phase. If there is residue, appropriate heating to 45 °C and extended concentration time can be considered and selected. A total of 100 μL of MeOH was added into the centrifuge tube for resuspending and swirled in ThermoMixer^®^C for 30 min (2000 rpm, 30 °C). Centrifugation was conducted at 15,000 rpm and 4 °C for 10 min, and 50 μL of the supernatant was taken into the injection bottle for measurement. Since the next step is that the extraction liquid will be directly fed into the chromatographic column for analysis, it is necessary to pay strict attention to the aspiration specification when aspirating the supernatant, and it is necessary to aspirate close to the liquid surface to prevent the chromatographic column from clogging due to the sucking of residual solids at the bottom of the centrifuge tube into the sample bottle. Refer to Figure 5 for the specific process. In particular, since 7-DHC is a substance that is susceptible to auto-oxidation and photodecomposition [87], we should completely protect ourselves from light to minimize the loss of 7-DHC when performing sample collection, oxysterol extraction, and sample preparation.

### 4.5. LC-MS/MS and Characterization of Oxysterols

**Figure 6:**
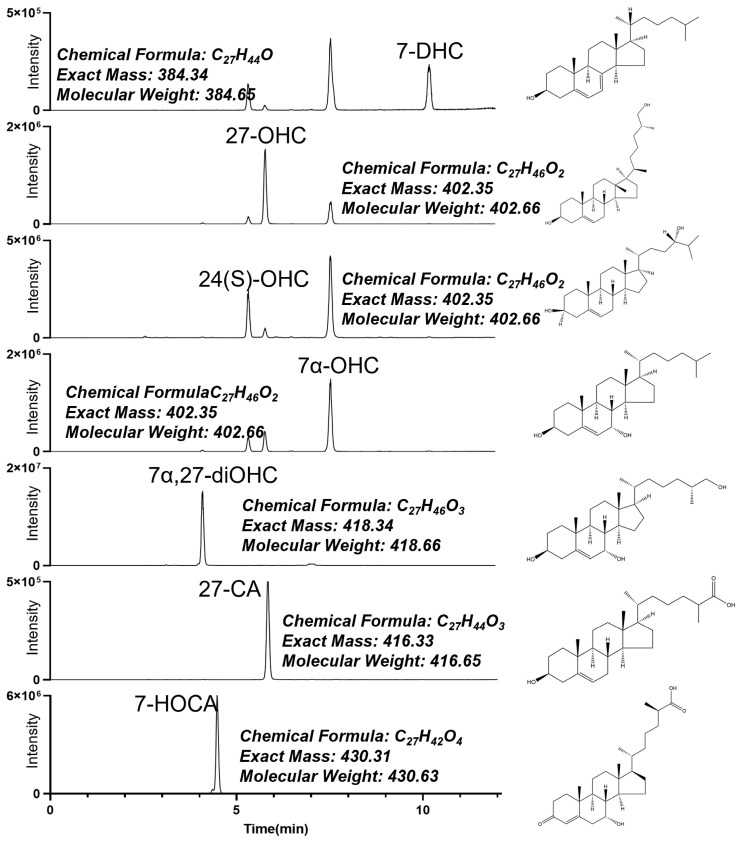
Retention time and structure information of oxysterols over the chromatogram. 27-OHC, 27-hydroxycholesterol; 24(S)-OHC, 24(S)-hydroxycholesterol; 7α-OHC, 7α-hydroxycholesterol; 7α,27-diOHC, 7α,27-dihydroxycholesterol; 7-DHC, 7-dehydrocholesterol; 27-CA, 3-hydroxy-5-cholestenoic acid; 7-HOCA, 7α-hydroxy-3-oxo-4-cholestenoic acid. Due to the presence of isomers in oxysterols, it is possible to have two or more peaks in one chromatogram.

LC-MS/MS analysis was conducted on a 1290 Infinity ultra-performance liquid chromatography (UPLC) system (Santa Clara, CA, USA) coupled with an Applied Biosystems/MDS Sciex 6500QTRAP mass spectrometer (Framingham, MA, USA). We conducted the chromatographic separation of oxysterols on Agilent InfinityLab Poroshell 120 phenyl-hexyl column (2.1 × 100 mm, 2.7 μm) with a flow rate of 0.3 mL/min. The column temperature was set to 30 °C, and the injection volume was 3 μL. The mobile phase was deionized water containing 0.3% formic acid (A) and methanol (B). Gradient elution was summarized as follows: 0~1 min, 20%~80% B; 1~9 min, 80%~90% B; 9~11 min, 90%~95% B; 11~11.01 min, 95%~20% B; 11.01~12 min, 20% B. The total analysis time required 12 min, and more polar compounds were diverted to waste for the first 1 min to keep the mass spectrometer clean. The ionization mode was OptiFlow™ Turbo V™ electro-spray ionization (ESI). Analytes were monitored by multiple reaction monitoring (MRM), which optimized quantifier or qualifier transitions are listed in Table 10. The structural information for each analyte is shown in Figure 6. Data acquisition and processing were conducted using AB SCIEX Analyst version 1.6.3. and MultiQuant Software version 3.0.3.

### 4.6. Method Validation

To validate the LC-MS/MS method, we evaluated the linearity, sensitivity, recovery, precision, matrix effect, repeatability, and stability.

Standard curves were calculated by measuring the seven oxysterols in triplicate using a linear least-squares regression model, and the R^2^ was used to evaluate linearity. In order to evaluate the repeatability of this method when determining extremely low concentration samples, based on the average concentrations of oxysterols in plasma, cerebral cortex and liver, clinical detection requirements and instrumentation, we simulate samples of 27-OHC, 24(S)-OHC, 7α-OHC, 7-DHC and 27-CA at 5 ng/mL in blood, 7α, 27-diOHC and 7-HOCA at 1 ng/mL in the cerebral cortex, and 24(S)-OHC at 100 ng/mL in the cerebral cortex. Six samples of the same concentration were configured for detection and CVs were recorded. LLOD is defined as the concentration of the lowest detectable analyte with a signal-to-noise ratio (S/N) of 3:1 and LLOQ is defined as the concentration of the lowest quantifiable analyte with a S/N of 10:1, and both are determined for each standard [88]. To evaluate apparent recovery, working solutions were added to biological samples to produce LQC, MQC, and HQC solutions, which were processed using the “oxysterol extraction” method. The six repetitive preparations of each concentration were made. The apparent recovery was ascertained by computing the ratio of the discrepancy between the QC sample calculated concentration and the actual added concentration. LQC, MQC, and HQC working solutions were repeatedly prepared in biological samples. QC working solutions were examined three times daily for three consecutive days to measure their intra-day and inter-day precision. Matrix effect (ME) pertains to the influence exerted by the substrate in a biological sample on the signal of the measured object. The 100 μL biological samples and the six aliquots of 100 μL methanol were prepared, respectively. After extraction by the MTBE system, 20 μL of LQC, MQC, and HQC working solutions were added to the supernatant and mixed in a vortex shaker. We calculated the mean value fm of QC in biological samples and the mean value fs of QC in methanol. ME% = fm/fs × 100%. ME% should range from 85% to 115%. Stability experiments were carried out using MQC samples. The stability was evaluated by testing the posttreatment MQC samples at 4 °C and 25 °C for 0 h, 4 h, 12 h, and 24 h, respectively.

## Figures and Tables

**Table 1:** Optimization of chromatographic conditions.

Parameter	Condition
Source Temperature	550 °C
Left Temperature	30 °C
Right Temperature	30 °C
Nebulizer Current	5 μA
Flow Rate	0.3 mL/min
Ion Sorce Gas 1	55 psi
Ion Sorce Gas 2	55 psi
Curtain Gas	35 psi
IonSpray Voltage	5500 V
Polarity	Positive

**Table 2:** Linearity of various oxysterols.

Analytes	Matrix	Retention Time (min)	Regression Equation	R^2^	LLOQ (ng/mL)	LLOD (ng/mL)
27-OHC	Plasma	5.47	y = 0.73770x − 0.00420	0.9981	4.31	1.29
	Cortex		y = 0.70269x + 0.04706	0.9998	2.06	0.62
	Liver		y = 0.50937x + 0.04004	0.9999	4.28	1.28
24(S)-OHC	Plasma	5.07	y = 1.54301x − 0.00902	0.9976	2.96	0.89
	Cortex		y = 1.45445x + 25.25826	0.9997	6.10	1.83
	Liver		y = 3.36508x + 0.20754	0.9998	2.59	0.78
7α-OHC	Plasma	7.30	y = 5.70410x + 3.96581	0.9952	3.01	0.90
	Cortex		y = 4.84027x + 7.79806	0.9999	0.50	0.15
	Liver		y = 9.81160x + 8.90440	0.9998	1.14	0.34
7α,27-diOHC	Plasma	3.92	y = 6.82848x + 0.96761	0.9990	1.98	0.59
	Cortex		y = 8.05137x − 0.12994	0.9988	0.56	0.17
	Liver		y = 9.64172x + 0.90577	0.9979	1.61	0.48
7-DHC	Plasma	9.64	y = 0.28292x + 0.19774	0.9961	3.23	0.97
	Cortex		y = 0.12466x + 1.01116	0.9998	2.06	0.62
	Liver		y = 0.40376x + 0.52205	0.9998	10.12	3.04
7-HOCA	Plasma	4.26	y = 1.96473x + 0.09463	0.9999	2.14	0.64
	Cortex		y = 3.49432x + 0.09240	0.9997	0.63	0.19
	Liver		y = 4.75955x − 0.36010	0.9998	2.58	0.78
27-CA	Plasma	5.50	y = 0.40085x + 0.00381	0.9991	3.91	1.17
	Cortex		y = 0.37399x − 0.02340	0.9991	3.20	0.96
	Liver		y = 0.46822x + 0.03005	0.9999	2.22	0.67

LLOQ, lower limit of quantitation; LLOD, lower limit of detection; 27-OHC, 27-hydroxycholesterol; 24(S)-OHC, 24(S)-hydroxycholesterol; 7α-OHC, 7α-hydroxycholesterol; 7α,27-diOHC, 7α,27-dihydroxycholesterol; 7-DHC, 7-dehydrocholesterol; 27-CA, 3-hydroxy-5-cholestenoic acid; 7-HOCA, 7α-hydroxy-3-oxo-4-cholestenoic acid.

**Table 3:** Intra-day or inter-day precisions of oxysterol analysis.

Analytes	Matrix	LQC Intra-Day			Inter-Day	MQC Intra-Day			Inter-Day	HQC Intra-Day			Inter-Day
		Day 1	Day 2	Day 3		Day 1	Day 2	Day 3		Day 1	Day 2	Day 3	
		CV (%)											
27-OHC	Plasma	6.84	1.09	1.80	4.39	9.82	7.32	5.86	7.86	2.10	0.71	1.28	3.52
	Cortex	8.61	5.26	9.12	7.88	1.67	3.12	1.38	2.24	3.34	1.24	4.13	3.34
	Liver	10.79	5.45	4.42	7.64	3.17	2.14	4.14	5.38	3.05	1.83	2.61	4.98
24(S)-OHC	Plasma	4.49	3.69	9.08	6.33	5.98	9.21	8.48	8.02	6.44	2.52	2.73	6.84
	Cortex	3.36	2.34	3.76	4.79	1.54	1.06	0.96	2.80	10.70	4.42	1.95	7.25
	Liver	5.14	6.17	3.53	5.54	5.24	3.96	8.63	6.94	3.89	1.84	3.16	4.03
7α-OHC	Plasma	2.43	7.08	0.78	8.52	10.37	8.36	7.83	9.80	5.44	3.68	2.34	5.18
	Cortex	7.12	7.98	4.43	7.26	6.17	3.70	2.15	5.61	4.41	1.12	3.08	5.13
	Liver	5.60	4.91	1.42	4.71	1.44	2.12	1.38	2.62	3.84	1.54	1.23	6.27
7α,27-diOHC	Plasma	2.81	5.97	6.90	6.16	5.15	5.42	5.27	5.90	1.93	3.73	1.64	3.70
	Cortex	3.77	4.38	4.37	4.44	2.12	3.42	3.03	3.02	4.68	2.04	3.66	3.99
	Liver	2.14	8.00	7.63	8.00	1.84	1.31	2.42	4.55	5.33	1.34	2.95	4.06
7-DHC	Plasma	8.50	8.10	3.80	9.11	7.82	7.36	2.81	9.01	4.91	6.03	4.55	6.51
	Cortex	8.31	1.90	9.36	9.96	4.81	5.82	4.46	6.98	3.11	5.13	4.82	5.27
	Liver	4.82	3.07	9.79	9.14	7.51	6.40	8.70	7.98	4.89	1.06	1.80	5.05
7-HOCA	Plasma	0.47	2.64	2.08	8.59	3.42	7.14	8.77	7.82	1.14	1.02	4.75	6.68
	Cortex	2.69	5.97	2.49	4.18	0.73	1.77	2.34	1.97	3.39	2.54	3.14	3.86
	Liver	2.17	3.02	4.58	4.43	1.02	1.26	1.89	1.54	2.76	1.96	1.17	3.04
27-CA	Plasma	5.31	7.98	5.13	7.98	9.32	7.35	5.12	9.04	8.57	4.45	2.32	6.18
	Cortex	3.65	4.52	4.83	4.68	2.32	3.50	2.54	2.95	4.08	3.12	3.85	4.42
	Liver	4.34	1.64	5.31	4.98	1.86	5.23	4.02	5.72	3.37	1.51	1.47	3.16

CV, coefficient of variation; 27-OHC, 27-hydroxycholesterol; 24(S)-OHC, 24(S)-hydroxycholesterol; 7α-OHC, 7α-hydroxycholesterol; 7α,27-diOHC, 7α,27-dihydroxycholesterol; 7-DHC, 7-dehydrocholesterol; 27-CA, 3-hydroxy-5-cholestenoic acid; 7-HOCA, 7α-hydroxy-3-oxo-4-cholestenoic acid; LQC, low-quality control; MQC, mid-quality control; HQC, high-quality control.

**Table 4:** Recovery of oxysterols analysis.

Analytes	Matrix	LQC (%)	CV (%)	MQC (%)	CV (%)	HQC (%)	CV (%)
27-OHC	Plasma	109.79	3.10	103.68	6.49	112.32	2.49
	Cortex	100.77	6.02	109.45	2.14	111.54	2.06
	Liver	93.70	3.94	102.70	3.15	104.69	1.77
24(S)-OHC	Plasma	100.91	6.70	109.61	2.86	108.70	2.75
	Cortex	90.85	2.85	101.88	8.63	95.03	3.51
	Liver	96.97	9.77	104.54	6.72	90.86	2.30
7α-OHC	Plasma	96.52	7.76	101.18	9.43	104.05	6.64
	Cortex	91.04	4.23	98.00	4.83	92.51	6.46
	Liver	101.13	5.08	92.70	3.45	95.41	8.70
7α,27-diOHC	Plasma	97.10	8.11	103.99	5.94	108.14	2.52
	Cortex	109.98	3.40	110.38	1.18	106.47	3.74
	Liver	88.47	3.86	100.26	3.62	92.88	1.66
7-DHC	Plasma	100.96	9.37	90.433	4.24	105.5	5.12
	Cortex	99.94	6.40	99.00	6.90	105.29	3.49
	Liver	91.17	3.54	100.30	9.57	97.17	7.43
7-HOCA	Plasma	98.85	7.61	97.45	9.53	104.35	4.62
	Cortex	107.62	3.52	106.16	3.42	109.13	3.99
	Liver	89.18	1.86	88.97	2.44	90.12	2.24
27-CA	Plasma	108.62	3.19	94.43	8.77	90.21	3.13
	Cortex	104.43	5.96	108.96	2.38	110.38	3.51
	Liver	94.75	5.98	100.65	3.78	97.76	1.65

LQC, low-quality control; MQC, mid-quality control; HQC, high-quality control; CV, coefficient of variation, 27-OHC, 27-hydroxycholesterol; 24(S)-OHC, 24(S)-hydroxycholesterol; 7α-OHC, 7α-hydroxycholesterol; 7α,27-diOHC, 7α,27-dihydroxycholesterol; 7-DHC, 7-dehydrocholesterol; 27-CA, 3-hydroxy-5-cholestenoic acid; 7-HOCA, 7α-hydroxy-3-oxo-4-cholestenoic acid. Measurements were expressed as percentages, and measurement accuracy was quantified using CV.

**Table 5:** Matrix effect of oxysterols analysis.

Analytes	Matrix	LQC ME (%)	MQC ME (%)	HQC ME (%)
27-OHC	Plasma	103.89	105.21	100.45
	Cortex	111.64	87.58	97.00
	Liver	110.28	95.22	96.67
24(S)-OHC	Plasma	98.12	111.63	103.72
	Cortex	99.02	111.88	98.93
	Liver	113.79	95.37	101.22
7α-OHC	Plasma	107.75	91.81	107.99
	Cortex	107.84	96.03	99.27
	Liver	87.91	105.99	87.57
7α,27-diOHC	Plasma	90.09	101.14	94.76
	Cortex	91.17	101.01	100.16
	Liver	108.63	85.24	114.57
7-DHC	Plasma	99.96	87.84	114.76
	Cortex	113.54	96.44	100.24
	Liver	85.74	93.23	102.81
7-HOCA	Plasma	87.88	85.60	93.47
	Cortex	105.07	96.71	92.96
	Liver	90.97	88.15	86.96
27-CA	Plasma	106.17	92.00	99.76
	Cortex	98.14	114.47	85.26
	Liver	103.23	103.67	107.53

ME, matrix effect; LQC, low-quality control; MQC, mid-quality control; HQC, high-quality control; CV, coefficient of variation, 27-OHC, 27-hydroxycholesterol; 24(S)-OHC, 24(S)-hydroxycholesterol; 7α-OHC, 7α-hydroxycholesterol; 7α,27-diOHC, 7α,27-dihydroxycholesterol; 7-DHC, 7-dehydrocholesterol; 27-CA, 3-hydroxy-5-cholestenoic acid; 7-HOCA, 7α-hydroxy-3-oxo-4-cholestenoic acid.

**Table 6:** Repeatability and stability of oxysterols analysis.

Analytes	Matrix	Background (ng/mL)	Target Conc. (ng/mL)	Calculated Conc. (ng/mL)	CV (%)	4 °C CV (%)	25 °C CV (%)
27-OHC	Plasma	0	5	5.35 ± 0.27	2.50	4.65	3.75
	Cortex	4.20	5	9.93 ± 1.00	5.03	2.92	2.65
	Liver	7.11	5	11.94 ± 2.20	9.21	8.07	4.62
24(S)-OHC	Plasma	0	5	5.18 ± 0.52	5.02	5.53	5.64
	Cortex	160.39	100	256.06 ± 18.57	7.25	9.56	9.95
	Liver	0	5	4.96 ± 065	6.58	7.88	4.33
7α-OHC	Plasma	0	5	4.85 ± 0.68	7.04	7.15	8.86
	Cortex	0	5	5.36 ± 0.34	6.29	5.87	8.11
	Liver	0	5	4.96 ± 0.74	7.48	9.64	5.80
7α,27-diOHC	Plasma	0	5	4.98 ± 0.74	7.43	3.41	2.83
	Cortex	0.89	1	1.85 ± 0.29	7.77	8.48	10.16
	Liver	0	5	4.87 ± 0.76	7.75	5.97	5.21
7-DHC	Plasma	0	5	5.14 ± 0.86	8.38	7.71	8.65
	Cortex	0	5	5.22 ± 0.80	7.65	7.58	7.60
	Liver	52	5	55.59 ± 6.28	5.65	8.45	6.49
7-HOCA	Plasma	0	5	4.63 ± 0.37	4.01	7.19	3.91
	Cortex	1.43	1	2.38 ± 0.15	6.48	5.51	5.09
	Liver	6.29	5	10.21 ± 0.69	3.35	2.61	2.24
27-CA	Plasma	0	5	5.31 ± 0.23	2.13	1.55	7.18
	Cortex	4.29	5	9.42 ± 1.12	5.92	5.78	3.96
	Liver	0	5	4.83 ± 0.90	9.36	4.23	4.05

CV, coefficient of variation; Target Conc., target concentration; Calculated Conc., calculated concentration; 27-OHC, 27-hydroxycholesterol; 24(S)-OHC, 24(S)-hydroxycholesterol; 7α-OHC, 7α-hydroxycholesterol; 7α,27-diOHC, 7α,27-dihydroxycholesterol; 7-DHC, 7-dehydrocholesterol; 27-CA, 3-hydroxy-5-cholestenoic acid; 7-HOCA, 7α-hydroxy-3-oxo-4-cholestenoic acid. Calculated Conc. was presented as mean ± SD.

**Table 7:** Oxysterols concentrations of mouse plasma.

Analytes	Matrix	Mean (ng/mL)	SD (ng/mL)
27-OHC	Plasma	10.74	4.48
24(S)-OHC	Plasma	35.14	14.50
7α-OHC	Plasma	45.53	19.51
7α,27-diOHC	Plasma	<0.01	<0.01
7-DHC	Plasma	305.23	104.13
7-HOCA	Plasma	26.35	17.76
27-CA	Plasma	10.47	3.76

SD, standard deviation; 27-OHC, 27-hydroxycholesterol; 24(S)-OHC, 24(S)-hydroxycholesterol; 7α-OHC, 7α-hydroxycholesterol; 7α,27-diOHC, 7α,27-dihydroxycholesterol; 7-DHC, 7-dehydrocholesterol; 27-CA, 3-hydroxy-5-cholestenoic acid; 7-HOCA, 7α-hydroxy-3-oxo-4-cholestenoic acid.

**Table 8:** Oxysterols concentrations of mouse cerebral cortex and liver.

Analytes	Matrix	Mean (ng/mg)	SD (ng/mg)
27-OHC	Cortex	0.40	0.15
	Liver	0.58	0.26
24(S)-OHC	Cortex	32.38	7.71
	Liver	0.06	0.01
7α-OHC	Cortex	0.24	0.29
	Liver	<0.01	<0.01
7α,27-diOHC	Cortex	0.04	0.01
	Liver	<0.01	<0.01
7-DHC	Cortex	7.44	2.88
	Liver	2.83	2.07
7-HOCA	Cortex	<0.01	<0.01
	Liver	0.09	0.03
27-CA	Cortex	0.06	<0.01
	Liver	0.45	0.21

SD, standard deviation; 27-OHC, 27-hydroxycholesterol; 24(S)-OHC, 24(S)-hydroxycholesterol; 7α-OHC, 7α-hydroxycholesterol; 7α,27-diOHC, 7α,27-dihydroxycholesterol; 7-DHC, 7-dehydrocholesterol; 27-CA, 3-hydroxy-5-cholestenoic acid; 7-HOCA, 7α-hydroxy-3-oxo-4-cholestenoic acid. The unit of oxysterols in the cerebral cortex and liver tissue is ng/mg. 7-HOCA and 27-CA are predominantly distributed in the periphery and are, therefore, almost undetected at the center.

**Table 9:** Concentration gradients of oxysterols.

Analytes	Matrix	Concentration Gradients (ng/mL)						
		STD1	STD2	STD3	STD4	STD5	STD6	STD7
27-OHC	Plasma	5	10	25	50	250	500	1000
	Cortex	5	10	25	50	250	500	1000
	Liver	5	10	25	100	250	500	1000
24(S)-OHC	Plasma	5	10	25	50	250	500	1000
	Cortex	100	250	500	1000	2500	5000	10,000
	Liver	5	10	25	50	100	500	1000
7α-OHC	Plasma	5	10	25	50	250	500	1000
	Cortex	5	10	25	50	250	500	1000
	Liver	5	25	50	100	250	500	1000
7α,27-diOHC	Plasma	5	10	25	50	250	500	1000
	Cortex	1	5	25	50	250	500	1000
	Liver	5	10	50	100	250	500	1000
7-DHC	Plasma	5	10	25	50	250	500	1000
	Cortex	5	10	25	50	250	500	1000
	Liver	10	25	50	100	250	500	1000
7-HOCA	Plasma	5	10	25	50	250	500	1000
	Cortex	1	5	10	25	50	250	1000
	Liver	5	10	25	50	100	250	1000
27-CA	Plasma	5	10	25	50	250	500	1000
	Cortex	5	10	25	50	250	500	1000
	Liver	5	10	25	50	100	500	1000

27-OHC, 27-hydroxycholesterol; 24(S)-OHC, 24(S)-hydroxycholesterol; 7α-OHC, 7α-hydroxycholesterol; 7α,27-diOHC, 7α,27-dihydroxycholesterol; 7-DHC, 7-dehydrocholesterol; 27-CA, 3-hydroxy-5-cholestenoic acid; 7-HOCA, 7α-hydroxy-3-oxo-4-cholestenoic acid.

**Table 10:** MRM parameters for oxysterols.

Analyte	Quantifier Transition	Qualifier Transition	DP (V)	CE (V)
27-OHC	385.4–>161.1	385.4–>135.0	160	27, 28
24(S)-OHC	385.4–>367.4	385.4–>135.2	110	15, 35
7α-OHC	385.4–>367.3	385.4–>159.0	60	19, 32
7α,27-diOHC	401.4–>383.2	401.4–>159.0	50	18, 32
7-DHC	367.5–>159.2	367.5–>145.3	80	24, 20
7-HOCA	431.1–>395.5	431.1–>413.2	130	25, 25
27-CA	399.2–>105.0	399.2–>81.0	100	46, 55
27-OHC-D5	390.5–>161.1	390.5–>135.0	100	25, 30
24-OHC-D7	392.5–>374.5	392.5–>255.2	100	15, 25

DP, declustering potential; CE, collision energy. 27-OHC, 27-hydroxycholesterol; 24(S)-OHC, 24(S)-hydroxycholesterol; 7α-OHC, 7α-hydroxycholesterol; 7α,27-diOHC, 7α,27-dihydroxycholesterol; 7-DHC, 7-dehydrocholesterol; 27-CA, 3-hydroxy-5-cholestenoic acid; 7-HOCA, 7α-hydroxy-3-oxo-4-cholestenoic acid; 27-OHC-D5, 27-hydroxycholesterol-26,26,26,27,27-d5; 24-OHC-D7, 24-hydroxycholesterol-25,26,26,26,27,27,27-d7. For each oxysterol targeted, the table contains precursor m/z values, fragments selected for transitions, fragment m/z values, optimized collision energies, and declustering potential.

## Data Availability

The data presented in this study are available on request from the corresponding author due to privacy.
